# Complete Genome Sequence of Stenotrophomonas maltophilia Myophage Moby

**DOI:** 10.1128/MRA.01422-19

**Published:** 2020-01-02

**Authors:** Alison Vicary, Heather Newkirk, Russell Moreland, Carlos F. Gonzalez, Mei Liu, Jolene Ramsey, Justin Leavitt

**Affiliations:** a Center for Phage Technology, Texas A&M University, College Station, Texas, USA; DOE Joint Genome Institute

## Abstract

Stenotrophomonas maltophilia is a prevalent nosocomial pathogen with multidrug resistance. Here, we describe the complete genome of S. maltophilia myophage Moby, which shares characteristics with Enterobacteria phage T4 and is closely related to Stenotrophomonas phage IME-SM1. Moby has a 159,365-bp genome with 271 predicted protein-coding genes and 24 predicted tRNAs.

## ANNOUNCEMENT

Stenotrophomonas maltophilia is a multidrug-resistant Gram-negative bacterium with rising prevalence as a nosocomial pathogen (1–3). Although it is a commensal found in diverse environments, including water, rhizospheres, and animal microflora, S. maltophilia carries natural and acquired antibiotic resistance genes (1, 2). As it is a human pathogen—in particular, one of the most common pathogens isolated from the lungs of cystic fibrosis patients—there is interest in finding phages for possible therapeutic use (1–3). Here, we present the annotated genome of S. maltophilia myophage Moby.

Moby was isolated from filtered (0.2-μm pore size) wastewater collected in Bryan, TX. The S. maltophilia host (ATCC 17807) was grown aerobically at 30°C in nutrient broth or agar (BD), and the phage was propagated by the soft-agar overlay method (4). Phage DNA was purified with a modified Promega Wizard DNA clean-up system shotgun library preparation protocol (5). An Illumina TruSeq library prepared with the Nano low-throughput kit was sequenced using Illumina MiSeq v2 500-cycle chemistry with paired-end 250-bp reads. On the 844,502 total reads in the phage-containing index, quality control analysis was done with FastQC (www.bioinformatics.babraham.ac.uk/projects/fastqc), and trimming was performed using FastX Toolkit v0.0.14 (http://hannonlab.cshl.edu/fastx_toolkit/). Assembly into a single contig with 673.7-fold coverage was performed using SPAdes v3.5.0 (6). The genome was confirmed complete by Sanger sequencing of the PCR product amplified off the contig ends (forward, 5′-CCTCGACAAGAAGAGGAGATTC-3′; reverse, 5′-CCTCGTCAAACATCTGGTTACT-3′). GLIMMER v3.0 and MetaGeneAnnotator v1.0 were used to predict protein-coding genes (7, 8). tRNA genes were predicted with ARAGORN v2.36 (9). Rho-independent termination sites were annotated using TransTermHP v2.09 (10). Gene functions were predicted based on analyses with InterProScan v5.33-72, TMHMM v2.0, and LipoP v1.0 and on BLAST v2.2.31 searches against NCBI nonredundant, UniProtKB Swiss-Prot, and TrEMBL databases with a 0.001 maximum expectation value (11–15). Structural predictions were done with HHSuite v3.0 tool HHPred (multiple sequence alignment generation with HHblits using the ummiclus30_2018_08 database and modeling with the PDB_mmCIF70 database) (16). progressiveMauve v2.4.0 was used to calculate genome-wide DNA sequence similarity (17). All tools are hosted in the Galaxy and Web Apollo instances hosted by the Center for Phage Technology (https://cpt.tamu.edu/galaxy-pub/), and, unless otherwise stated, were executed using default parameters (18, 19). Phage morphology was observed by transmission electron microscopy of samples negatively stained with 2% (wt/vol) uranyl acetate at the Texas A&M Microscopy and Imaging Center (20).

Myophage Moby has a 159,365-bp double-stranded DNA genome with a coding density of 93.0% and a G+C content of 54.1%, which is lower than the 66.7% G+C content of its host (21). The genome encodes 271 predicted protein-coding genes, 73 of which were assigned putative functions, and 24 tRNA genes. PhageTerm analysis predicted permuted termini, consistent with the *pac*-type headful DNA packaging mechanism used by T4-like phages (22). Moby shares 196 similar proteins and 92.29% nucleotide identity with *Stenotrophomonas* phage IME-SM1 (GenBank accession no. KR560069), an unclassified myovirus. No introns were identified in Moby, including in the predicted thymidylate synthase and *nrdB* genes, which contain introns in phage T4 (GenBank accession no. NC_000866) (23).

### Data availability.

The genome sequence and associated data for phage Moby were deposited under GenBank accession no. MN095772, BioProject accession no. PRJNA222858, SRA accession no. SRR8893605, and BioSample accession no. SAMN11414490.
